# Prevalence, etiology and antibiotic resistance patterns of community-acquired urinary tract infections in Dhaka, Bangladesh

**DOI:** 10.1371/journal.pone.0274423

**Published:** 2022-09-15

**Authors:** Mohammad Aminul Islam, Md Rayhanul Islam, Rizwana Khan, Mohammed Badrul Amin, Mahdia Rahman, Muhammed Iqbal Hossain, Dilruba Ahmed, Muhammad Asaduzzaman, Lee W. Riley

**Affiliations:** 1 Paul G. Allen School for Global Health, College of Veterinary Medicine, Washington State University, Pullman, Washington, United States of America; 2 Laboratory of Food Safety and One Health, Laboratory Sciences and Services Division, International Centre for Diarrhoeal Disease Research, Bangladesh (icddr,b), Dhaka, Bangladesh; 3 Clinical Microbiology Laboratory, Laboratory Sciences and Services Division, International Centre for Diarrhoeal Disease Research, Bangladesh (icddr,b), Dhaka, Bangladesh; 4 Centre for Global Health, Institute of Health and Society, University of Oslo, Oslo, Norway; 5 Division of Infectious Diseases and Vaccinology, School of Public Health, University of California, Berkeley, Berkeley, CA, United States of America; Tribhuvan University, NEPAL

## Abstract

Urinary tract infection (UTI) accounts for a significant morbidity and mortality across the world and is a leading cause for antibiotic prescriptions in the community especially in developing countries. Empirical choice of antibiotics for treatment of UTI is often discordant with the drug susceptibility of the etiologic agent. This study aimed to estimate the prevalence of community-acquired UTI caused by antibiotic resistant organisms. This was a cross-sectional study where urine samples were prospectively collected from 4,500 patients at the icddr,b diagnostic clinic in Dhaka, Bangladesh during 2016–2018. Urine samples were analyzed by standard culture method and the isolated bacteria were tested for antibiotic susceptibility by using disc diffusion method and VITEK-2. Descriptive statistics were used to estimate the prevalence of community acquired UTI (CA-UTI) by different age groups, sex, and etiology of infection. Relationship between the etiology of CA-UTI and age and sex of patients was analyzed using binary logistic regression analysis. Seasonal trends in the prevalence of CA-UTI, multi-drug resistant (MDR) pathogens and MDR *Escherichia coli* were also analyzed. Around 81% of patients were adults (≥18y). Of 3,200 (71%) urine samples with bacterial growth, 920 (29%) had a bacterial count of ≥1.0x10^**5**^ CFU/ml indicating UTI. Women were more likely to have UTI compared to males (OR: 1.48, CI: 1.24–1.76). *E*. *coli* (51.6%) was the predominant causative pathogen followed by *Streptococcus* spp. (15.7%), *Klebsiella* spp. (12.1%), *Enterococcus* spp. (6.4%), *Pseudomonas* spp. (4.4%), coagulase-negative *Staphylococcus* spp. (2.0%), and other pathogens (7.8%). Both *E*. *coli* and *Klebsiella* spp. were predominantly resistant to penicillin (85%, 95%, respectively) followed by macrolide (70%, 76%), third-generation cephalosporins (69%, 58%), fluoroquinolones (69%, 53%) and carbapenem (5%, 9%). Around 65% of patients tested positive for multi-drug resistant (MDR) uropathogens. A higher number of male patients tested positive for MDR pathogens compared to the female patients (*p* = 0.015). Overall, 71% of Gram-negative and 46% of Gram-positive bacteria were MDR. The burden of community-acquired UTI caused by MDR organisms was high among the study population. The findings of the study will guide clinicians to be more selective about their antibiotic choice for empirical treatment of UTI and alleviate misuse/overuse of antibiotics in the community.

## Introduction

Urinary tract infections (UTIs) in both community and hospital settings are estimated to affect around 405 million people globally and nearly 0.23 million people died of UTIs, contributing to 5.2 million disability-adjusted life years (DALYs) in 2019 [1]. Treatment of UTI begins empirically often with different broad-spectrum antibiotics [2]. The incidence of UTIs caused by multidrug-resistant uropathogens has been increasing at an alarming rate worldwide. Such common infections can turn into life-threatening illnesses, especially in developing countries [3, 4]. The frequency, spectrum, and antibiotic resistance in uropathogens differ according to geographical locations and time, which necessitates a thorough understanding of the epidemiology of community-acquired UTIs (CA-UTIs) [5].

The predominant organism causing both complicated and uncomplicated UTI is uropathogenic *Escherichia coli* followed by *Klebsiella pneumoniae*, *Enterococcus faecalis*, *Proteus mirabilis*, and group B *Streptococcus* (GBS) [6]. Further, multi-drug resistant *E*. *coli* and *K*. *pneumoniae* are increasingly recognized to cause both CA-UTIs and hospital-acquired UTIs [7, 8]. Because of these two pathogens, empirical treatment for acute pyelonephritis frequently involves a third-generation cephalosporin [9]. However, inappropriate use of antibiotics is very common in the management of CA-UTI. In developing countries, the scenario is debilitating as a significant proportion of UTI patients purchase antibiotics directly from community pharmacies without prescription or any expert consultation [10, 11]. Consequently, common antibiotics become ineffective. Although UTI can occur in any age and sex groups, female children, women in their reproductive age, and older women are more vulnerable to infections [12].

UTI is a significant public health problem in Bangladesh, which accounts for considerable morbidity [13], health care cost [14] due to frequent treatment failure, and recurrent infections. UTI is also one of the main reasons for misuse of antibiotics in the community leading to the escalating burden of antimicrobial resistance [11]. A study carried out in 2012 among 443 suspected UTI patients in a regional medical college hospital in Bangladesh showed that 43% of patients had significant bacterial growth of uropathogens in their urine samples [15]. A recent study has shown that more than 75% of *E*. *coli* causing UTI are resistant to third-generation cephalosporin [16]. Existing reports are based on either a small sample size, targeting only a specific population or age group, analysis of retrospective data from hospital registry, or characterization of convenience samples of bacterial isolates obtained from urine samples of UTI patients [17, 18]. The proportion of multi-drug resistant uropathogens is likely to have increased over time. There is no large-scale prospective survey of UTIs in Bangladesh that can provide an up-to-date information on the burden of infections in the community. Continuous monitoring of the etiology of infections and antibiotic resistance pattern is essential not only for selecting appropriate antibiotics for empirical therapy but also for reducing the overuse/misuse of antibiotics. We carried out a community-based study to investigate the etiologies of UTI along with antibiotic resistance profiles for 2 years in Dhaka, Bangladesh.

## Methods

### Ethical approval

All participants provided written informed consent before taking part in the study. Written informed consent was obtained from the parents/guardians of the minors (<16 years of age) included in this study. The research team explained the background and objectives of the study clearly to the participants. This study was approved by the Institutional Review Board (IRB) of icddr,b (protocol number: PR 16071).

### Study design

This study is a cross-sectional survey of patients from communities who visited the clinical diagnostic center of the International Centre for Diarrhoeal Disease Research, Bangladesh (icddr,b) from 1^st^ September 2016 to 30^th^ November 2018. The icddr,b clinical diagnostic center serves as reference laboratories for clinical diagnostic analysis of human diseases cohorts and control subjects. Approximately 1500 patients and individuals avail diagnostic services each day from the diagnostic outpatient department. The diagnostic laboratories are the only accredited laboratories under ISO15189 (quality) and ISO15190 (safety) in Bangladesh for as many as 160 different tests and parameters. The diagnostic center only offers on-payment clinical diagnostic services and does not provide any consultancy services to the patient. During the study period, patient’s consents were taken every day from 9 am to 5 pm except for the weekends and government holidays. Study physicians approached only those patients at the diagnostic center who ordered for urine culture and sensitivity analysis and requested for their willingness to allow the study team to use culture-sensitivity results of their urine sample and use the resulting bacterial isolates for further characterization. In Bangladesh, it is not required to show physician’s order to the diagnostic clinics for urine culture and sensitivity test and patients can order for the test by themselves. We did not assess whether they had any urinary tract complaints or had any symptoms of UTI but we used the following exclusion criteria for enrolment of the patients: 1) patients having any medical or surgical device in their bodies (e.g., catheter, cannula) 2) patients having renal stone 3) patients admitted to hospital. Enrolled patients were segregated into three age groups: adults (≥18y), adolescents (11-17y) and children (0-10y).

### Sample collection and processing

Clean catch midstream urine was collected in a sterile urine container by the patient or by the caregiver of the patient (in case of children). Samples from the consented patients were labeled with a green color sticker containing our study name for tracking. Urine culture was done in the clinical microbiology lab of icddr,b following standard procedure within 1 h of collection [19]. Briefly, a loopful (~10μl) of urine samples were streaked on MacConkey agar (Oxoid Ltd., UK) and blood agar (Oxoid Ltd., UK) by a semi-quantitative method using a calibrated loop. Plates were incubated at 35°C aerobically and examined at 18–24 hours and they were further incubated for another 24 hours before a negative report was issued. Cultures were quantitated, and ‘significant’ bacteriuria was defined as a case of UTI in specimen containing bacterial species ≥1.0x10^5^ CFU/ml of urine according to the standard guideline [20]. Patients with non-significant bacteriuria (<1.0x10^5^ CFU/ml) were excluded from further analysis.

### Identification of bacterial species

Isolates obtained from 1^st^ September 2016 to 17^***th***^ December 2017 were identified based on Gram’s stain reaction, culture characteristics and biochemical properties as described earlier [21], while the isolates obtained from 18^***th***^ December 2017 to 30^th^ November 2018 were identified using VITEK-2 system.

### Antibiotic susceptibility tests

Antibiotic susceptibility testing of uropathogens isolated during the period from 1^st^ September 2016 to 17^th^ December 2017 was performed by the disk diffusion method following the Clinical and Laboratory Standards Institute (CLSI) guidelines using commercially available antibiotic disks (Oxoid Ltd., UK) [22]. Susceptibility testing for isolates obtained between 18^th^ December 2017 to 30^th^ November 2018 was done with VITEK 2 system. Antibiotic susceptibility tests were performed against 37 commonly prescribed antibiotics from 13 different classes of which aminoglycoside (gentamycin 10μg), macrolide (azithromycin 15μg), cephalosporins (ceftriaxone 30 μg; cefotaxime 30μg; ceftazidime 30μg; cefepime 30μg; cefixime 5μg), penicillin (ampicillin 10μg), penicillin/beta lactamase inhibitor combination(piperacillin-tazobactam 110μg), phenicols (chloramphenicol 30μg), tetracycline (tetracycline 30 μg), linezolid (linezolid 30μg), (fluoro)quinolone (nalidixic acid 30μg; ciprofloxacin 5μg), carbapenem (imipenem 10μg, meropenem, 10μg), nitrofurans (nitrofurantoin 300μg), glycylcyclines (vancomycin 30μg) and sulfonamides (sulfamethoxazole-trimethoprim 25μg) were tested against both Gram-positive and Gram-negative bacteria. In addition, polymyxin (polymyxin-B 300μg) and glycopeptide (vancomycin 30μg) antibiotics were tested only against Gram-negative and Gram-positive organisms, respectively. Isolates were categorized as sensitive, intermediate, or resistant according to the CLSI guideline. All third-generation cephalosporin-resistant *E*. *coli* isolates were tested for ESBL production by combination disk diffusion test as described by CLSI using cefotaxime (CTX 30 μg), ceftazidime (CAZ, 30 μg) alone and in combination with clavulanic acid (CLA, 10 μg) [22]. Isolates were considered as ESBL-producer if the inhibition zone diameter was 5 mm larger with CLA than without [22]. If an organism was resistant to at least one agent in three or more classes of antibiotics, it was categorized as multidrug resistant (MDR) [23]. All MDR *E*. *coli* isolates were subcultured on fresh MacConkey plates and isolated colonies were stored at -80°C for further analysis.

### Data analysis

We entered data into SPSS 20.0 (IBM Inc., Chicago, USA). Data cleaning, statistical analyses, and data visualization were done in Stata 13.0 (College Station, Texas, USA) and MS Office Excel 2010 (Microsoft, Washington, USA). Descriptive statistics including frequency distribution, percentage, and cross-tabulation were done among different age groups, sex, and etiology of infection. Percent of resistance to different antibiotics and resistance to multiple antibiotics in each organism was determined. Data were analyzed to determine the association between the proportion of samples that tested positive for MDR, non-MDR, third-generation cephalosporin-resistant, and carbapenem-resistant organisms, with age and sex by Chi-square test and Fisher exact test where applicable. Binary logistic regression was done by considering a sample positive for MDR as a dependent variable and age, sex as independent variables. Seasonal trends in the prevalence of CA-UTI, MDR pathogens, MDR, and non-MDR *E*. *coli* were analyzed to see the changing patterns of infection over two years (September 2016-November 2018). Statistical significance was determined at alpha = 0.05 for all tests.

## Results

### Prevalence of community acquired UTIs

During the period from September 2016 to November 2018, around 39,000 patients visited the icddr,b diagnostic center in Dhaka for doing culture and sensitivity analysis of their urine samples. In this study we enrolled 4,500 patients of which 3,643 (81%) were adults and 3,263 (73%) were females. Of these urine samples, 3,200 (71%) tested positive for microbial growth on culture plates, of which 920 (29%) had a significant bacterial count (≥1.0x10^5^ CFU/ml of urine), defined as cases of UTI (Table 1). According to this criterion, the prevalence of CA-UTI cases among samples tested was 20% (920 of 4,500). The prevalence of CA-UTI was significantly higher among adult patients compared to adolescents and children (OR: 1.86, CI: 1.48–2.34) (Table 2). The prevalence of CA-UTI was also significantly higher among female patients (OR: 1.48, CI: 1.24–1.76) (Table 2). However, age-adjusted female patients and sex-adjusted age showed a lower risk of CA-UTI than the crude rate (Table 2).

**Table 1:** Characteristics of patients according to their age, sex and urine culture status.

Covariates	Male (N = 1,237) n (%)	Female (N = 3,263) n (%)	Total (N = 4,500) n (%)
**Age group**			
Adult **(**18 & above**)**	895 (72)	2,748 (84)	3,643 (81)
Adolescent (11–17)	48 (4)	95 (3)	143 (3)
Child (0–10)	294 (24)	420 (13)	714 (16)
**Growth status**			
Growth positive	747 (60)	2,453 (75)	3,200 (71)
Significant growth (≥10^5^ CFU/ml of urine)	199 (27)	721 (29)	920 (29)
Insignificant (<10^5^ CFU/ml of urine)	548 (73)	1,732 (71)	2,280 (71)
No growth	477 (39)	720 (22)	1,197 (27)
Collection Contamination (≥ 3 organisms)	13 (1)	90 (3)	103 (2)

**Table 2:** Prevalence of community-acquired UTI among patients according to different age and sex groups.

Characteristics	No (%) of UTI patients	Age (Mean±SD)	COR (CI)	AOR (CI)
**Age**				
Adult (n = 3,643)	808 (22)	45.96±16.11	1.86 (1.48,2.34)	1.76 (1.40,2.22)
Adolescent (n = 143)	17 (12)	14.33±4.56	0.88 (0.51,1.52)	0.86 (0.49, 1.49)
Child (n = 714)	95 (13)	4.70±5.25	1.00	1.00
**Sex**				
Male (n = 1,237)	199 (16)	37.00±24.43	1.00	1.00
Female (n = 3,263)	721 (22)	38.95±20.18	1.48 (1.24,1.76)	1.39 (1.17, 1.66)

COR, crude odds ratios; AOR, adjusted odds ratios, CI, confidence intervals, SD, standard deviation

### Etiologies of CA-UTI

**Fig 1:**
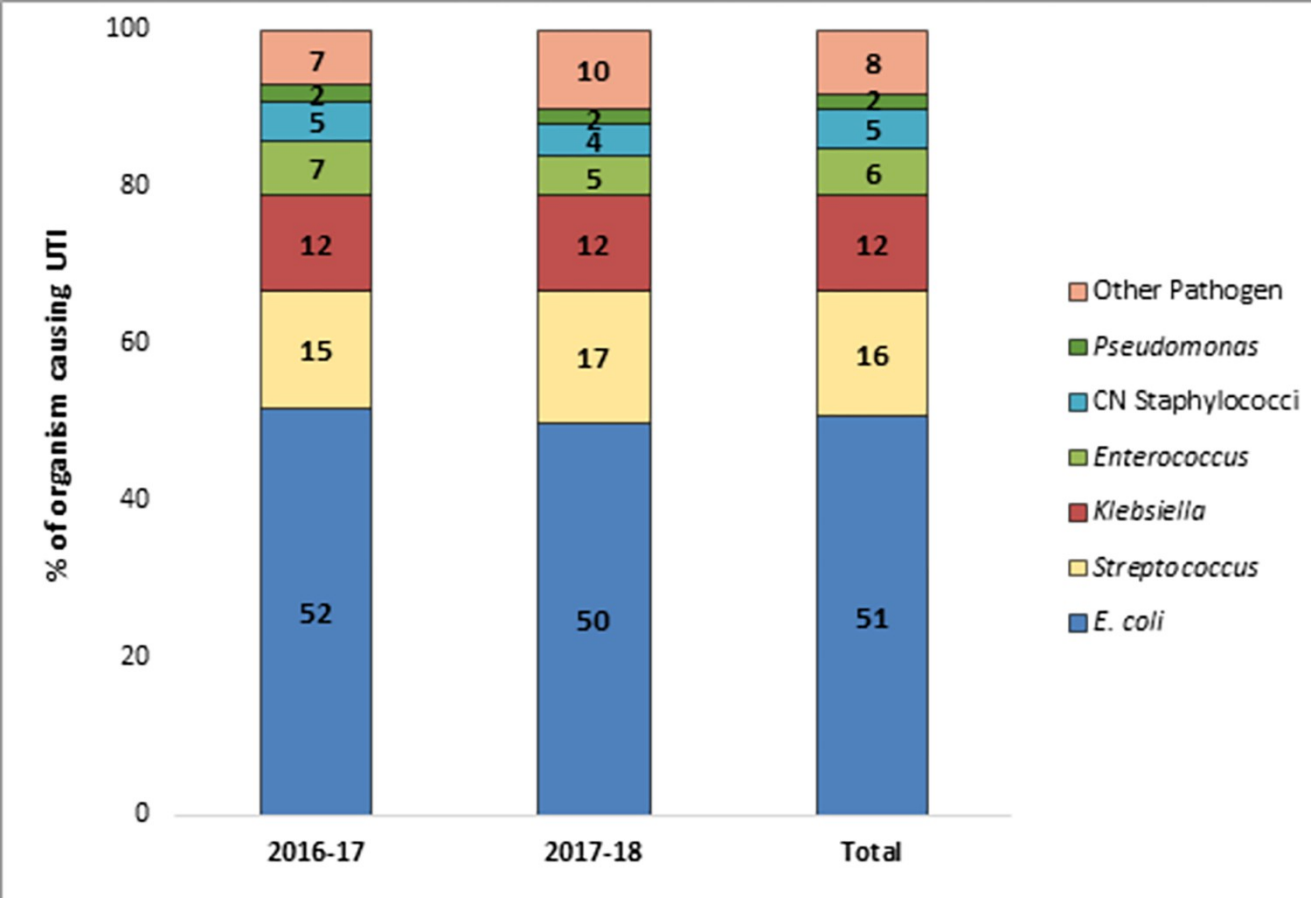
Prevalence of major bacterial pathogens causing community-acquired UTI among patients from 2016 to 2018.

Culture of urine samples from 920 patients yielded significant growth of uropathogens including 849 (92%) monomicrobial (single bacterial species) and 71 (8%) polymicrobial (pair of two different bacterial species) growths. A total of 991 [849+ (71*2)] isolates were obtained from 920 patients of which 989 were bacterial isolates and 2 were identified as *Candida* spp. Among bacterial isolates, *E*. *coli* was the predominant (51.6%), followed by *Streptococcus* spp. (15.7%), *Klebsiella* spp. (12.1%), *Enterococcus* spp. (6.4%), *Pseudomonas* spp. (4.4%), coagulase negative *Staphylococcus* spp. (2.0%), *Enterobacter* spp. (1.8%), *Proteus* spp. (1.6%), *Acinetobacter* spp. (1.0%), *Staphylococcus saprophyticus* (1.1%), *Staphylococcus aureus* (0.6%), *Corynebacterium* spp. (0.3%), *Serratia* spp. (0.3%), and *Sphingomonas paucimobilis* (0.3%). A significant proportion of *Streptococcus* spp. and *Enterococcus* spp. were *Streptococcus agalactiae* (52%), and *Enterococcus faecalis* (59%), respectively. Due to some limitations of conventional culture-based methods in identifying organisms at species level we report the prevalence of some organisms at genus level. Distribution of isolates over two years showed almost similar trends with *E*. *coli* being the predominant organism in year 1 (52%) and year 2 (50%), followed by *Streptococcus* spp. (15%, 17%, respectively), *Klebsiella* spp. (12%, 12%), *Enterococcus* spp. (7%, 5%), coagulase negative *Staphylococcus* spp. (5%, 4%), *Pseudomonas* spp. (2%, 2%), and other pathogens (7%, 10%) (Fig 1). Of 71 polymicrobial infections, 39 (55%) tested positive for only Gram-negative bacteria, 17 (24%) tested positive for only Gram-positive bacteria and 15 (21%) tested positive for a mix of Gram-positive and Gram-negative bacteria. The predominant pair of organisms (13 of 71) included lactose fermenting and non-fermenting *E*. *coli* followed by a pair of *E*. *coli* and *Klebsiella* spp.

### Antibiotic resistance patterns of uropathogens

**Fig 2:**
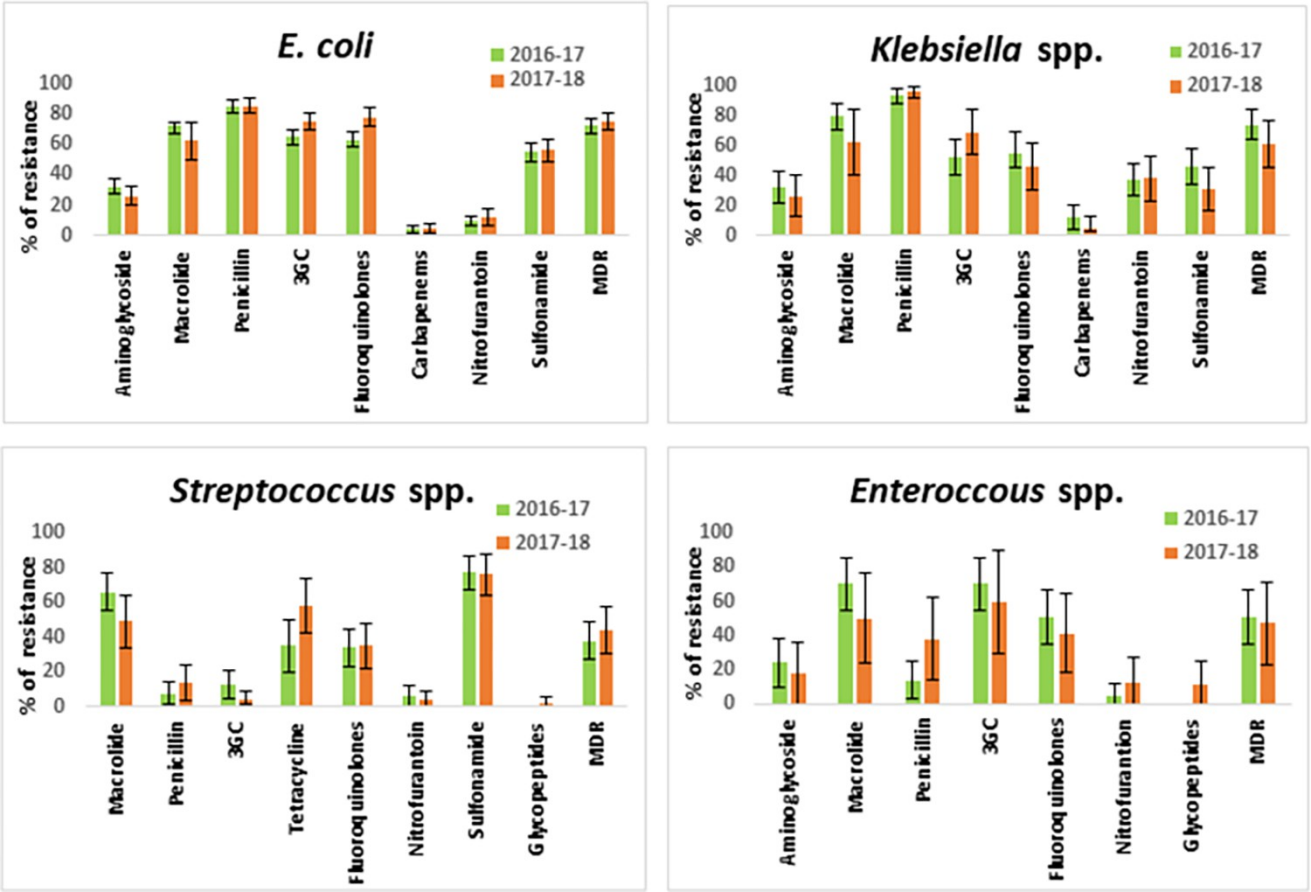
Antibiotic resistance profiles of four major pathogens causing community-acquired UTI from 2016 to 2018. Only those antibiotics were included in the analysis for which the minimum number of isolates tested was 10.

Among Gram-negative bacteria, *E*. *coli* and *Klebsiella* spp. were resistant to different classes of antibiotics with the highest rate of resistance to penicillin (85%, 95%, respectively) followed by macrolide (70%, 76%), third-generation cephalosporins (69%, 58%), fluoroquinolone (69%, 53%) and sulfonamide (56%, 41%). The difference in the prevalence of resistance in *E*. *coli* and *Klebsiella* spp. was significant for several antibiotics including third-generation cephalosporins, fluoroquinolones, nitrofurantoin, and sulphonamide (Fig 2). For both *E*. *coli* and *Klebsiella* spp. no significant variation in resistance frequency was observed between the two years (Fig 2). Carbapenem resistance was the least prevalent in both *E*. *coli* (5%) and *Klebsiella* spp. (9%) (Fig 2). Overall, 454 (71%) Gram-negative isolates were multidrug-resistant and *E*. *coli* and *Klebsiella* spp. were the predominant with a prevalence of 74% and 68%, respectively. Of 510 *E*. *coli* isolates, 350 (69%) were third-generation cephalosporins resistant (3GCr) and 233 (67%) of 3GCr *E*. *coli* were positive for ESBL.

Of 260 Gram-positive bacterial isolates, antibiotic susceptibility test results were available for 194 (75%) isolates. Among the prevalent Gram-positive organisms, both *Streptococcus* spp. and *Enterococcus* spp. were predominantly resistant to macrolide (59%, 65%, respectively). Only a small proportion (9%) of *Streptococcus* spp. was resistant to third-generation cephalosporin (Fig 2). Overall, 46% of Gram-positive isolates were identified as MDR, *Streptococcus* spp. and *Enterococcus* spp. were predominant with a prevalence of 37% and 35%, respectively.

### Distribution of uropathogens according to age and sex

**Fig 3:**
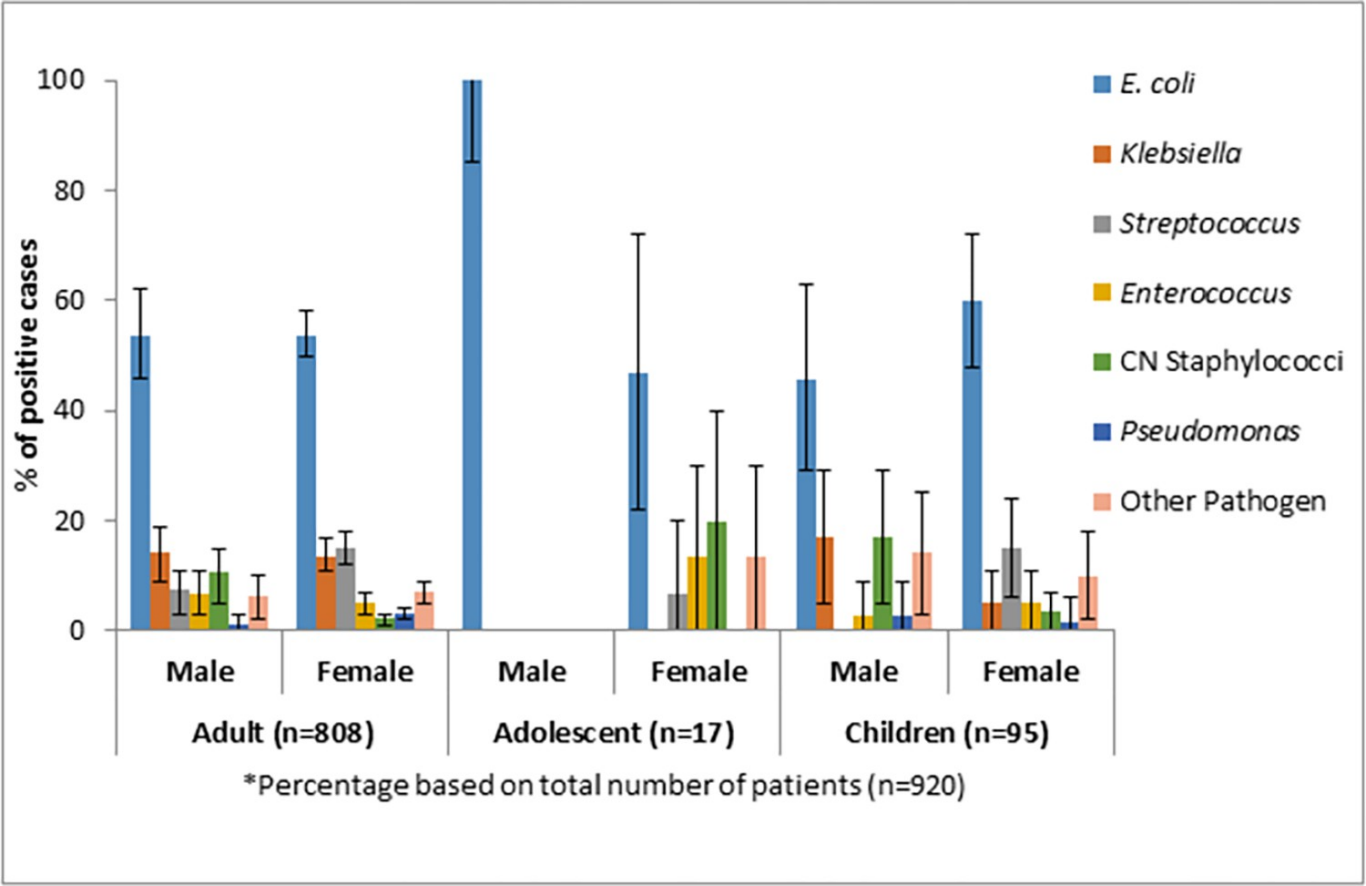
Distribution of the prevalence of major pathogens causing CA-UTI among patients by age and sex.

A significantly higher number of female patients (n = 721) had an infection with both Gram-negative and Gram-positive bacteria (*p*<001). Male to female ratio among laboratory-confirmed CA-UTI patients were 1:3 (age-adjusted odds ratio: 1.39, CI: 1.17–1.66). *E*. *coli* was the predominant uropathogen among adult female patients followed by *Streptococcus* spp., *Klebsiella* spp., and *Enterococcus* spp. We observed similar trends among adult male patients except that coagulase negative *Staphylococcus* spp. was more prevalent than *Enterococcus* spp. (Fig 3).

### Distribution of MDR uropathogens according to age and sex

The prevalence of MDR-UTIs did not differ significantly in patients of different age groups. However, a significantly higher proportion of male patients (73%) tested positive for MDR organisms compared to their female counterparts (63%) (*p* = 0.015). The difference was prominent among child patients. Male children had 72% (CI: 22%-90%) higher odds of being infected with MDR organisms compared to their female counterparts (Table 3). However, the difference was not statistically significant among patients positive for 3GCr and carbapenem resistant organisms at any age group, although higher proportions of male adult and child patients were positive for 3GCr organisms compared to their female counterparts (Table 4).

**Table 3:** Prevalence of MDR community-acquired UTI among patients according to different age and sex groups.

Covariates (n = 892)*	MDR (n = 575)	Age (yrs) (Mean±SD)	Non-MDR (n = 317)	Age (yrs) (Mean±SD)	OR (CI)
**Adult (n = 781)**	n (%)		n (%)		
Male (n = 157)	109 (69)	57.11**±**13.68	48 (31)	51.79**±**17.75	1
Female (n = 624)	393 (63)	51.99**±**15.33	231 (37)	46.25**±**16.37	0.75 (0.51, 1.09)
**Adolescent (n = 17)**					
Male (n = 2)	1 (50)	**-**	1 (50)	**-**	1
Female (n = 15)	9 (60)	13.18**±**2.41	6 (40)	14.33**±**3.32	1.50 (0.07, 28.89)
**Child (n = 94)**					
Male (n = 35)	29 (83)	2.66**±**2.66	6 (17)	1.42**±**1.24	1
Female (n = 59)	34 (58)	4.44**±**3.07	25 (42)	4.35±3.03	0.28 (0.10, 0.78)

*Antibiotic susceptibility results were available for 892 out of 920 patients

**Table 4:** Prevalence of 3GCr and carbapenem-resistant pathogens among patients with community-acquired UTI.

Covariates*	3GCr n (%)	Age (yrs) (Mean±SD)	OR (CI)	Covariates*	Carb^R^ n (%)	Age (yrs) (Mean±SD)	OR (CI)
**Adult (n = 521)**				**Adult (n = 521)**			
Male (n = 107)	77 (72)	58.88±12.97	1	Male (n = 107)	5 (5)	62.40±7.64	1
Female (n = 414)	271 (65)	53.27±14.17	0.74 (0.46–1.18)	Female (n = 414)	20 (5)	53.15±16.58	1.04 (0.38–2.83)
**Adolescent (n = 8)**				**Adolescent (n = 8)**			
Male (n = 2)	1 (50)	**-**	1	Male (n = 2)	1 (50)	**-**	**-**
Female (n = 6)	4 (67)	14.00±3.46	2 (0.07–51.59)	Female (n = 6)	0	**-**	**-**
**Child (n = 58)**				**Child (n = 58)**			
Male (n = 21)	15 (71)	2.00±1.85	1	Male (n = 21)	2 (10)	1.23±0.86	1
Female (n = 37)	24 (65)	4.67±3.03	0.74 (0.23–2.36)	Female (n = 37)	2 (5)	8±1.41	0.54 (0.07–4.17)

Carb^R^, carbapenem resistant; n, number; 3GCr, 3^rd^ generation cephalosporin resistant

*One isolate represents one patient. A total of 587 Gram-negative isolates were tested for susceptibility against third-generation cephalosporin and carbapenem antibiotics.

### Temporal distribution of antibiotic-resistant uropathogens among patients

We analyzed the distribution of antibiotic resistance in 991 isolates over two years to find the trends across years. There were no significant fluctuations in the prevalence of resistance to any particular antibiotic over the years among major uropathogens such as *E*. *coli*, *Klebsiella* spp., *Streptococcus* spp., and *Enterococcus* spp. (Fig 2). Overall, more than 50% of all Gram-negative isolates (range: 53% to 95%) were found consistently resistant to penicillin, third-generation cephalosporins and fluoroquinolones. Prevalence of ESBL-producing *E*. *coli* has significantly increased over the years (*p*<0.05).

## Discussion

Increased resistance to multiple antibiotics among pathogens causing CA-UTI leads to frequent treatment failure and complications. Drug-resistant CA-UTI has become a major global health problem, especially in developing countries like Bangladesh. However, longitudinal surveillance of CA-UTI estimating the burden of infection, etiology and antibiotic susceptibility patterns of uropathogens in Bangladesh is largely missing except for a few small observational studies among specific population groups such as pregnant women, children, and hospitalized patients [15, 17, 24, 25]. We conducted this study to fill this critical knowledge gap. Among 4,500 patients enrolled in this study during the 2 years, the prevalence of laboratory-confirmed CA-UTI cases (as defined by CFU ≥1.0x10^5^ CFU/ml from urine culture) was 20% with women bearing the greater burden (78%), similar to most studies of CA-UTI [26].

Although the majority of UTI cases were caused by a single pathogen, we found a small proportion of patients had polymicrobial growth in their urine culture mostly *E*. *coli* coupled with lactose non-fermenting *E*. *coli* or *Klebsiella* spp. In general, polymicrobial infections are less common in young, healthy, sexually active females [27]. In our study, we found that polymicrobial cases were predominant among female adult patients (52.3 ± 15 years), and there was no significant difference in prevalence among different age groups. Studies on polymicrobial cases of UTI in Bangladesh are limited, one study reported that 5% patient had polymicrobial growth in urine samples [15]. It is more likely that polymicrobial infections often go untreated or treated with inappropriate antibiotics leading to the development of antibiotic resistance in uropathogens [28].

Among monomicrobial cases, we found that *Streptococcus* spp. was the most prevalent cause of CA-UTI after *E*. *coli*, which is in agreement with a previous study among adult women in Dhaka slums [29]. However, two studies carried out with patients attending tertiary level hospitals outside of Dhaka (in Rajshahi and Mymensingh), reported that *Staphylococcus saprophyticus* was the second leading cause of UTI after *E*. *coli* [15, 18] while another study (in Comilla) reported *Klebsiella pneumoniae* as the second leading cause [30]. There might be some geographical variations in the etiology of UTI in Bangladesh, which might also be dependent on the age, sex, sexual activity (for female), clinical features and demographic characteristics of patients. However, irrespective of all variations among patients, *E*. *coli* appears to be the most common cause of UTI, and thus combating *E*. *coli* infections would largely reduce the burden of CA-UTI in the community. Among all *Streptococcus* spp. cases *S*. *agalactiae* or Group B *Streptococcus* (GBS) accounted for 52% (n = 80) and around 9% of the total number of patients with UTI; 92% (n = 74) of women were infected with GBS. In a previous study among pregnant women in rural Bangladesh, only a small proportion of (5.3%) patients were infected with GBS [24].

Around 65% of the patients in our study tested positive for an MDR pathogen comprising both Gram-positive and Gram-negative organisms, which is higher than the report from a neighboring country Nepal [31, 32]. There is scarcity of data on the overall prevalence of MDR pathogens in CA-UTI in Bangladesh and neighboring countries. The majority of studies focused on resistance patterns of the predominant bacterial pathogen causing UTI, such as *E*. *coli* or ESBL-producing *E*. *coli*. Among neighboring countries, a study in Kathmandu, Nepal reported that 41% of bacterial pathogens from UTI cases were MDR [32]. A similar study from Alighar, India reported that 42% of uropathogens were ESBL-producing, while another study from Pakistan showed that 66% of uropathogens were ESBL-producing [33]. Studies from India and Pakistan reported the occurrence of MDR *E*. *coli* as 43% and 59%, respectively [31, 34, 35]. In this study, we found that 74% of *E*. *coli* isolates were MDR and 69% of isolates were resistant to third-generation cephalosporins, which is higher than the prevalence of MDR *E*. *coli* (58%) reported earlier from Bangladesh [29]. Overall, 50% of all isolates were consistently resistant to macrolide, and penicillin, and 80% of isolates were sensitive to carbapenem, glycopeptides and rifampicin. In the case of *E*. *coli*, a significant proportion (70%) was sensitive to aminoglycosides, nitrofurantoin and carbapenem, which corroborates with data from other countries [36–40].

We found that male patients were more likely to be infected with MDR organisms than female patients. Similarly, prevalence of 3GCr and carbapenem-resistant organisms was higher among adult male compared to adult females. This finding correlates with the results of a large study conducted in Portugal where the prevalence of MDR uropathogens was higher in men than in women (35% vs. 22%) [41]. However, among adolescents and children, the difference in prevalence was not statistically significant. One of the reasons for the higher prevalence of MDR organisms among adult male in our study could be the older age as we found mean ages of the male adults with 3GCr and carbapenem-resistant infections were 58.9 (±13.0) and 62.4 (±7.6) years, respectively which is higher than the mean age for female adults with 3GCr (53.3±14.2) and carbapenem (53.1±16.6) resistant infections. Older males usually develop UTIs as a result of bladder outlet obstruction from prostate enlargement, which is often associated with a higher prevalence of resistance due to recurrence and prolonged antibiotic treatment with higher therapeutic doses [42, 43].

There were some limitations in our study. We carried out the surveillance among patients who came to icddr,b diagnostic facility to test their urine samples for culture and sensitivity analysis. A large proportion of patients receiving diagnostic services from icddr,b are the residents of Dhaka city and therefore the data may not be representative of the whole country. icddr,b diagnostic facility only provides clinical diagnostic services and does not offer any outpatient services and therefore, we could not estimate the proportion of patients with and without symptomatic infection. Nevertheless, our study provides a recent insight into the etiology of CA-UTI along with trends in antibiotic resistance among uropathogens over 2 years in a large sample size for the first time in Bangladesh.

## Conclusions

The burden of CA-UTI caused by MDR pathogens is high among the study participants and there was no significant difference in prevalence and etiology of infection over two years. The findings of the study can serve as an evidence base for updating guidelines for improved management of CA-UTI in Bangladesh and other developing countries with similar settings. It also provides a basis to conduct epidemiologic studies to investigate risk factors for the acquisition of antibiotic-resistant CA-UTI.

## Supporting information

S1 Data Data file for Figs 1–3 and the frequency of polymicrobial infections. (XLSX)

S1 File PLOS’ questionnaire on inclusivity in global research. (DOCX)

## List of abbreviations

UTI: Urinary tract infection
CA-UTIs: community-acquired UTIs
CLSI: Clinical and Laboratory Standards Institute
ESBL: extended spectrum beta-lactamase
MDR: multi-drug resistant
3GCr: third-generation cephalosporin-resistant
icddr,b: International Centre for Diarrhoeal Disease Research, Bangladesh
